# COVID-19 restrictions and NUTS-3 deprivation: multilevel approach in Italy during the second wave

**DOI:** 10.1093/eurpub/ckac129.461

**Published:** 2022-10-25

**Authors:** L Dei Bardi, A Acampora, M Di Martino, M Davoli, N Agabiti, G Cesaroni

**Affiliations:** 1 Department of Epidemiology of the Lazio Region, Rome, Italy; 2 Sapienza University of Rome, Rome, Italy

## Abstract

**Background:**

To face the second COVID-19 wave, Italy implemented a tiered restriction system with different risk levels (yellow=low; orange=medium, red=high). It is unknown whether the effect of the tiers was equal among provinces with varying levels of socioeconomic deprivation (SED). At each restriction level, we analyzed the impact of the province's SED on the SARS-CoV-2 daily reproduction number (Rt).

**Methods:**

We considered the Rt (Nov 2020-May 2021) as the dependent variable and the SED as the independent variable. The Rt was estimated using daily incidence data from the Civil Protection Department as the instantaneous Rt. The province SED was measured using the percentage of individuals whose yearly income was less than 10,000€ (2019 data from the Ministry of Economy and Finance). We used multilevel linear regression models with random intercepts stratified by restriction level to estimate the effect of the SED on Rt (β) and its Standard Error (SE). Our analyses adjusted the estimates for the number of days into the tier first and then for other covariates.

**Results:**

We found different levels and trends of Rt by SED in every restriction. Days-adjusted models found a containing effect for the red and the orange tier, while the Rt had an increasing trend in yellow. Higher SED was associated with higher Rt: β was positive and significant in red (β = 0.004 SE = 0.001) and orange (β = 0.002 SE = 0.001) but not in the lowest tier (β = 0.001 SE = 0.001). We found a significant interaction between the number of days into the restriction and the SED in the complete models. Compared to less deprived, more deprived provinces had slower Rt reduction in the highest tier. However, they had steeper Rt reductions in orange and slower increasing trends in yellow.

**Conclusions:**

The highest restriction had milder effects in more deprived provinces, while lower tiers were more effective. These results underline the importance of accounting for SED when implementing public health measures.

**Key messages:**

• Area-level deprivation can modify the effects of public health measures.

• Socioeconomic characteristics of the areas should be considered when implementing policies aimed to prevent the spread of epidemics.

